# Facial Pain due to Granulomatous Lesion in the Pterygopalatine Fossa

**DOI:** 10.1002/jgf2.70071

**Published:** 2025-10-10

**Authors:** Haruya Kawakami, Masahiro Biyajima, Wataru Ishii

**Affiliations:** ^1^ Department of General Internal Medicine Japanese Red Cross Nagano Hospital Nagano Japan

A 73‐year‐old man presented with persistent left‐sided facial pain for 1 month and a 4‐month history of chronic sinusitis. The pain, localized to the first and second branches of the left trigeminal nerve, was resistant to nonsteroidal anti‐inflammatory drugs, which had been used for 3 months without relief. He had no fever, weight loss, cold‐like illness, shingles, or other systemic symptoms. Past medical history included hyperthyroidism, varicose veins of the lower extremities, and inguinal hernia. Neurological examination was unremarkable except for localized tenderness in the distributions of the left trigeminal nerve. There was no saddle nose deformity. Laboratory testing showed elevated C‐reactive protein (6.85 mg/dL).

Noncontrast head CT revealed a soft‐tissue mass in the left pterygopalatine fossa along the course of the trigeminal nerve (Figure 1A), extending toward the foramen rotundum and inferior orbital fissure, consistent with possible involvement of the maxillary branch (V2). The chronic sinusitis was attributable to this mass lesion, and no other sinonasal lesions were detected on CT. Before biopsy, possible differential diagnoses included sarcoidosis, aspergillosis, tuberculosis, syphilis, and neoplasm. Biopsy showed granulation tissue extending into the adjacent bone, without evidence of vasculitis, giant cells, or granulomatous infection (Figure 1B). Myeloperoxidase‐ANCA was elevated at 77.4 U/mL, while PR3‐ANCA was negative. According to the Watts algorithm, chronic sinusitis persisting for more than 3 months was regarded as a surrogate marker, leading to the diagnosis of granulomatosis with polyangiitis (GPA) [1]. MRI showed no evidence of hypertrophic pachymeningitis, and there was no pulmonary or renal involvement.

**FIGURE 1:**
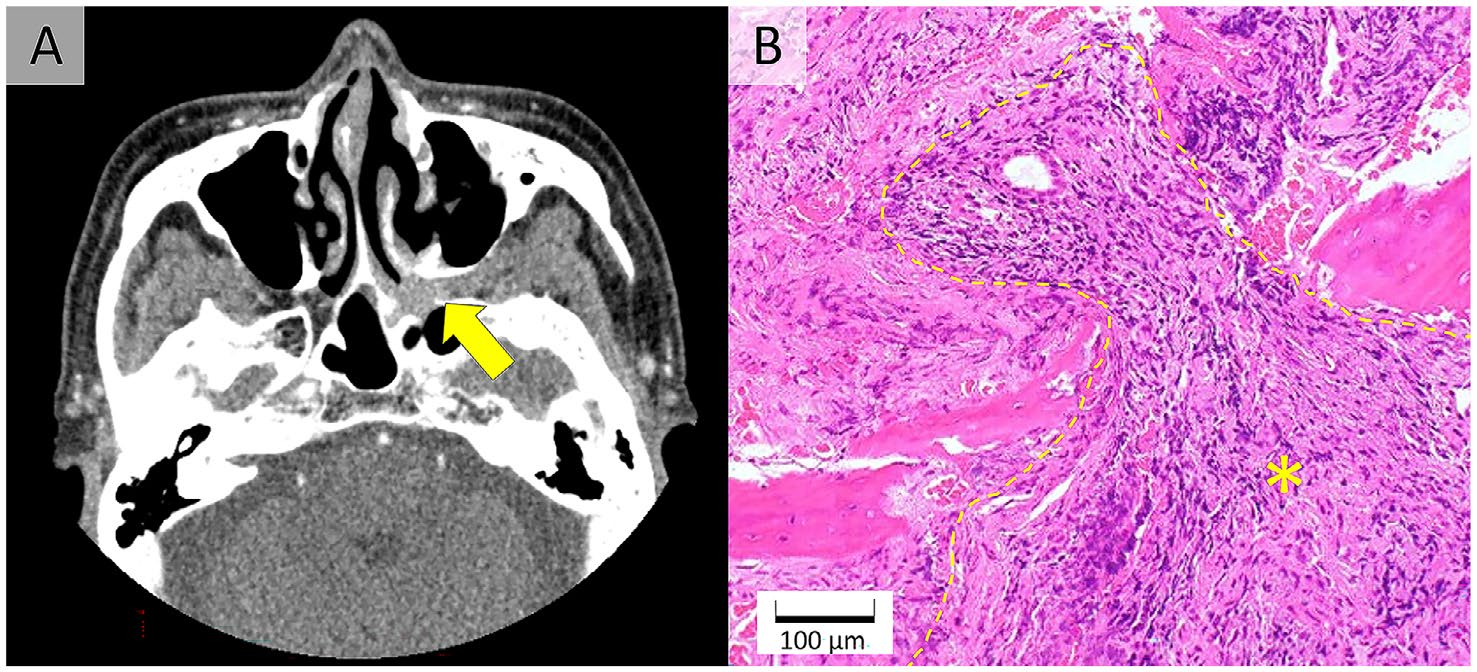
(A) Noncontrast head CT showing a soft‐tissue density in the left pterygopalatine fossa (arrow). (B) Histopathology reveals granulation tissue (*) extending into the adjacent bone.

Prednisolone (1 mg/kg/day) was initiated, resulting in marked improvement in facial pain within 3 months. Follow‐up imaging demonstrated regression of the pterygopalatine fossa lesion, confirming radiological as well as clinical improvement. While trigeminal nerve involvement is recognized in skull base GPA [2], isolated lesions in the pterygopalatine fossa are rare [3]. Our case highlights that GPA should be considered in the differential diagnosis of trigeminal neuralgia‐like facial pain, even in the absence of systemic features. Recognition of pterygopalatine fossa involvement, supported by histopathological findings and ANCA positivity, may help establish an early and accurate diagnosis. Careful imaging of the pterygopalatine fossa is essential for early detection and appropriate management.

## Author Contributions

Haruya Kawakami drafted the manuscript. Masahiro Biyajima and Wataru Ishii reviewed and revised the manuscript critically for important intellectual content. All authors were involved in the clinical care of the patient and approved the final version of the manuscript.

## Ethics Statement

The authors have nothing to report.

## Consent

Written informed consent for publication of this case report and the accompanying images was obtained from the patient. Patient anonymity has been preserved.

## Conflicts of Interest

The authors declare no conflicts of interest.

## Data Availability

All relevant data have been included in the report.
